# Alternative mapping of probes to genes for *Affymetrix *chips

**DOI:** 10.1186/1471-2105-5-111

**Published:** 2004-08-14

**Authors:** Laurent Gautier, Morten Møller, Lennart Friis-Hansen, Steen Knudsen

**Affiliations:** 1 Center for Biological Sequence Analysis, Technical University of Denmark, 2800 Lyngby, Denmark; 2 Dept. of Clinical Biochemistry, Rigshospitalet, University of Copenhagen, 2100 Copenhagen, Denmark

## Abstract

**Background:**

Short oligonucleotide arrays have several probes measuring the expression level of each target transcript. Therefore the selection of probes is a key component for the quality of measurements. However, once probes have been selected and synthesized on an array, it is still possible to re-evaluate the results using an updated mapping of probes to genes, taking into account the latest biological knowledge available.

**Methods:**

We investigated how probes found on recent commercial microarrays for human genes (Affymetrix HG-U133A) were matching a recent curated collection of human transcripts: the NCBI RefSeq database. We also built mappings and used them in place of the original probe to genes associations provided by the manufacturer of the arrays.

**Results:**

In a large number of cases, 36%, the probes matching a reference sequence were consistent with the grouping of probes by the manufacturer of the chips. For the remaining cases there were discrepancies and we show how that can affect the analysis of data.

**Conclusions:**

While the probes on Affymetrix arrays remain the same for several years, the biological knowledge concerning the genomic sequences evolves rapidly. Using up-to-date knowledge can apparently change the outcome of an analysis.

## Background

In a relatively short time microarrays have become a well established technique, widely used by researchers. Microarrays offer nothing less than to be able to monitor simultaneously the expression levels for thousands of genes.

The RNA molecules from the biological sample are called *targets*, and the polymers of nucleic acids fixed on the surface are called *probes*. The very large number of genes represented on each microarray requires the use of computer based approaches. Although such approaches currently constitute a very rich and active area of research, for many data analyses this step can be summarized simply: under the prior assumption that for the large majority of the genes represented on a microarray the expression will not vary significantly across experiments, the main focus is be to isolate the few genes of interest from the rest. Short oligonucleotide arrays are a particular type of microarrays. Short oligonucleotide arrays are constituted of short probes (oligonucleotides) with several probes designed to match different part of the target sequences. The use of techniques originating from the micro-electronics industry proved very successful in the making of short oligonucleotides arrays [1], helping the *Affymetrix *company establish itself as one of the primary manufacturers for microarrays. The pre-processing of oligonucleotide array data differs from other microarray data, specifically because probe intensities associated with each gene are generally summarized by an expression value, or expression index. As it contributes to the computation of the expression values, this step alone is of importance. Different algorithms have been suggested to replace and improve Affymetrix's original algorithms [2], including E. Lazaridis *et al*.'s *playerout *[3], Li and Wong's model [4], medianpolish [5], and Affymetrix's own improvements to its algorithms [6]. However, information about the individual probes was not disclosed until a few years ago. Only with the release of the probe sequences for a significant number of Affymetrix chips, data analysis approaches considering the nature of individual probes have been made possible. The use of the chemical nature of the probes, on which depends the binding energy with complementary sequences, has already been suggested to improve pre-processing of Affymetrix data at the probe level [7,8].

The annotation for sequenced genomes have progressed considerably since the design of the Affymetrix chips, even the most recent ones, and matching the latest transcriptomic (or genomic) data available with the chip designs is an obvious thing to do.

We have performed such a remapping for a few Affymetrix chips, and we show that the resulting probe-to-gene mapping can differ substantially from the original Affymetrix mapping. This can affect the interpretation of experimental data.

As annotations of genomes continue to evolve, it is also desirable to have a framework to perform and handle up-to-date probe-to-gene mapping. We provide an open source and documented implementation to do so.

## Results

The results obtained are subdivided in two main categories: the matches between probes and reference sequence obtained, and the difference in the outcome of an analysis when using an alternative mapping.

### Probes matching multiple RefSeq entries

**Figure 1:**
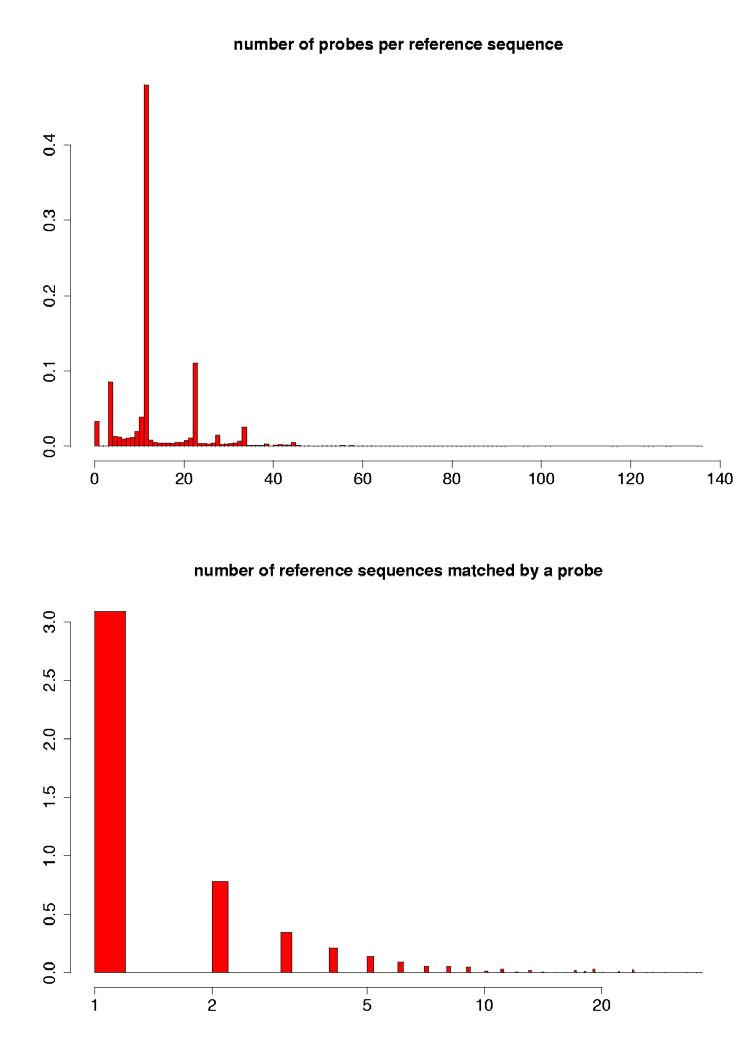
**Histrograms for the number of probes per probe set. **Both plots are histogram, the values on the y axis are relative frequencies. (top) Histogram of the number of probes on the U133A chip matching a particular RefSeq transcript. A clear peak can be seen for 11 probes, which corresponds to the number of probes in a probe set most commonly found in the *original mapping*. This set is called Alt1 in the last part of the section Results. (bottom) Histogram of number of RefSeq transcripts matching a single probe (log scale). Most of the probes can be seen matching only one RefSeq. In both plots, the probes associated to human 'ALU' repeats were filtered out.

A fair number of probes were found to match several reference sequences, as shown in Figure 1.

For example, the RefSeq *NM_001544.2 *is found to have 21 matching probes. Eleven of these matching probes also match another reference sequence: *NM_022377.1*. A quick look at the annotation reveals that both reference sequences are two different transcripts variants of the same gene 'Homo sapiens intercellular adhesion molecule 4, Landsteiner-Wiener blood group (ICAM4)' and that the same probes are found matching these two sequences. However the ten remaining probes matching *NM_001544.2 *are also found matching a fairly large number of other reference sequences (from a little less than 300 to almost 600 reference sequences, depending on the probe). We found that these probes are designed to match sub-sequences frequently found in mRNA: ALU repeats. All the probes matching ALU repeats are in the *official mapping *grouped in a common probe set, called 'human ALU'.

Besides 'human ALU' probes, other probes matching multiple reference sequences were found. In that case, the reference sequences matching a given probe have closely related annotations, or even identical annotations. Complex mixtures of partial overlaps for the probe sets can then be observed. As an example, the probe 88322 is found matching the reference sequences *NM_017445.1*, *NM_003519.3*, *NM_003520.3*, *NM_003521.2*, *NM_003525.2*, *NM_003528.2*, *NM_080593.1 *and *XM_301109.1*, annotated 'H2B histone family, member S (H2BFS), mRNA', 'histone 1, H2bl (HIST1H2BL), mRNA', 'histone 1, H2bn (HIST1H2BN), mRNA', 'histone 1, H2bm (HIST1H2BM), mRNA', 'histone 1, H2bi (HIST1H2BI), mRNA', 'histone 2, H2be (HIST2H2BE), mRNA', 'histone 1, H2bk (HIST1H2BK), mRNA' and 'similar to Histone H2B 291B (LOC350694), mRNA' respectively.

Such matches in each case require expert annotators to curate the *alternative mappings*, so we chose to simply ignore the probes matching several reference sequences in the rest of this study.

**Figure 2:**
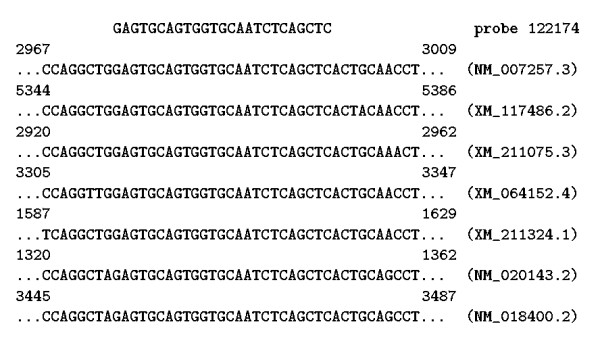
**Reference sequences matching the probe 122174. **The reference sequences matching the probe 122174, assigned to the probe set *211697_x_at *and annotated 'Homo sapiens RNA-binding protein LOC56902 mRNA, complete cds' in the *original mapping*. NCBI's reference sequences are annotated 'paraneoplastic antigen MA2 (PNMA2), mRNA', 'LOC202934 (LOC202934), mRNA', 'hypothetical protein LOC283507 (LOC283507), mRNA', 'sarcalumenin (SRL), mRNA', 'LOC284095 (LOC284095), mRNA', 'putative 28 kDa protein (LOC56902), mRNA' and 'sodium channel, voltage-gated, type III, beta (SCN3B), mRNA' respectively

Other multiple matches are more easy to handle, and potentially more harmful when included in an analysis. Some probes are found to hybridize to several unrelated reference sequences, as shown in Figure 2. Only 290 probes that were labeled mismatches in the *official *mapping, were found to be legitimate perfect match probes in the *alternative *mapping.

### Reference sequences matching all the probes from probe sets

**Figure 3:**
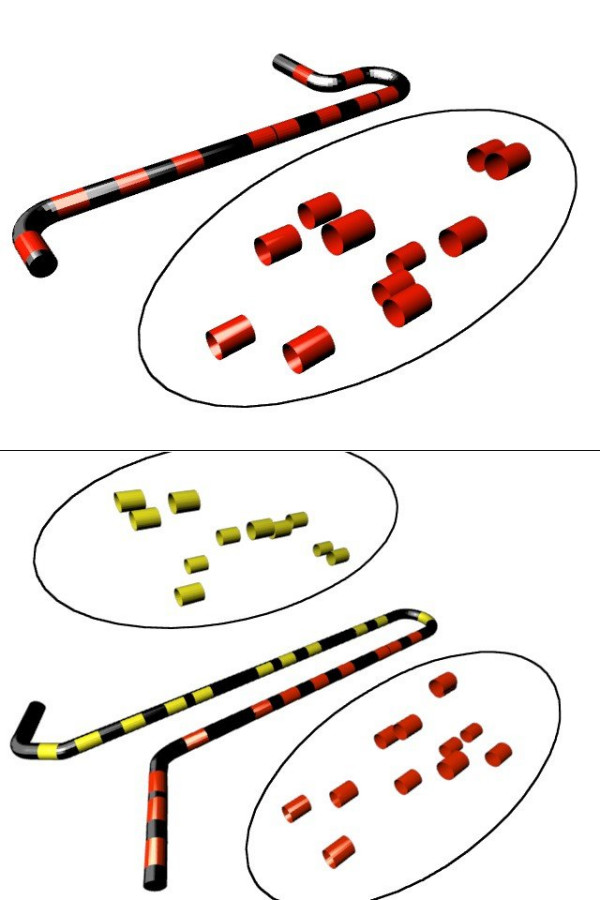
**Probes, probe sets and reference sequence. **Reference sequence matching all the probes from probe sets in the *official mapping*. The reference sequence is represented by a long dark cylinder, while the matching probes are represented by red or yellow fragments of cylinder. The wire frame represents the grouping of probes in a probe set in the *official mapping*. (Top:) All the probes matching the reference sequence constitute a probe set in the *official mapping*. (Bottom:) All the probes matching the reference sequence constitute two different probe sets in the *official mapping*.

We also found a significant number of reference sequences for which all the matching probes belong to one probe set in the *official mapping*. When a 'one to one' association can be established between a probe set of the *official mapping *and a probe set in an *alternative mapping*, which means that a given reference sequence matches all the probes associated with one *Affymetrix ID*, we conclude a complete agreement between the *alternative mapping *and the *official one *(See Figure 3, top). That is the case for 6274 out of 17426 reference sequences (17426 is the number of reference sequences for which at least one matching probe was found). When the association is 'one-to-many', in the sense that several complete probe sets in the *original mapping *are matching one reference sequence, one could conclude that, alternative splicing events left aside, some probe sets are redundant (See Figure 3, bottom).

**Figure 4:**
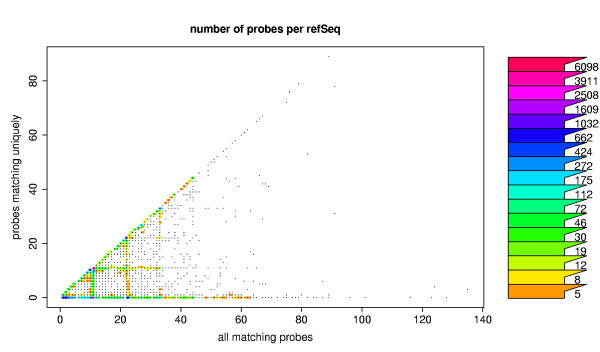
**Scatter plots for the number of probes matching a reference sequence. **Scatter plot of the total number of probes matching a reference sequence against the number of probes remaining after removing the probes matching several reference sequences. Colored areas are displayed to indicate the z-axis, the number of probes occupying each spot in the graph. A grid-like pattern can be observed in the lower-left corner of the plot. The size of the cells is 11 probes, which is the number of probes contained in most of the probe sets in the *official mapping*.

We obtain 1168, 212, 38, 4 and 2 reference sequences for which the matching probes are coming from 2, 3, 4, 5 and 6 *original *probe sets respectively. The Figure 4 shows that it represents a significant part of the cases. This represents 8% of the *original *probe sets of a HG-U133A that are potentially redundant.

### Effect on the outcome of an analysis

Naturally the expression values, or expression indexes, computed from the probes intensities are sensitive to differences in the mapping: different probes will give different summary expression values, which can have an effect on the outcome of an analysis. To verify it, we performed a standard exploratory analysis of Affymetrix data (two samples, looking for the genes that are significantly differentially expressed). The probe level intensities were pre-processed and expression values computed. The *original mapping *was used to obtain a first set of expression values (set Affy), while the *alternative mappings *made from matching NCBI's reference sequences was used to obtain two more sets of expression values (sets Alt1 and Set Alt2), using all the matching probes or all the probes matching uniquely respectively. In other words, the set Alt2 differs from the set Alt1 in the sense that probes in Alt1 matching several reference sequences were removed from Alt2. The set Affy describes 22283 probe sets, the set Alt1 describes 18076 probe sets (for a total of 184735 probes) and the set Alt2 11640 probe sets (for a total of 153257 probes). The number of mismatch probes in the *official mapping *that are found in the *alternative *mappings is low: 290 in Alt1 and 87 in Alt2.

For each one of the three sets, 'significantly differentially expressed genes' (SDEGs) are searched for: a Welch's two-sample t-test is performed on all the expression values in each set, and the selection for significant p-values done as described by Ventura and collaborators [9,10] (qvalue set to 1%). The number of SDEGs obtained in the sets Affy, Alt1 and Alt2 are 163, 163, and 103 respectively. Table 1 shows how many of these represent identical probe sets.

**Figure 5:**
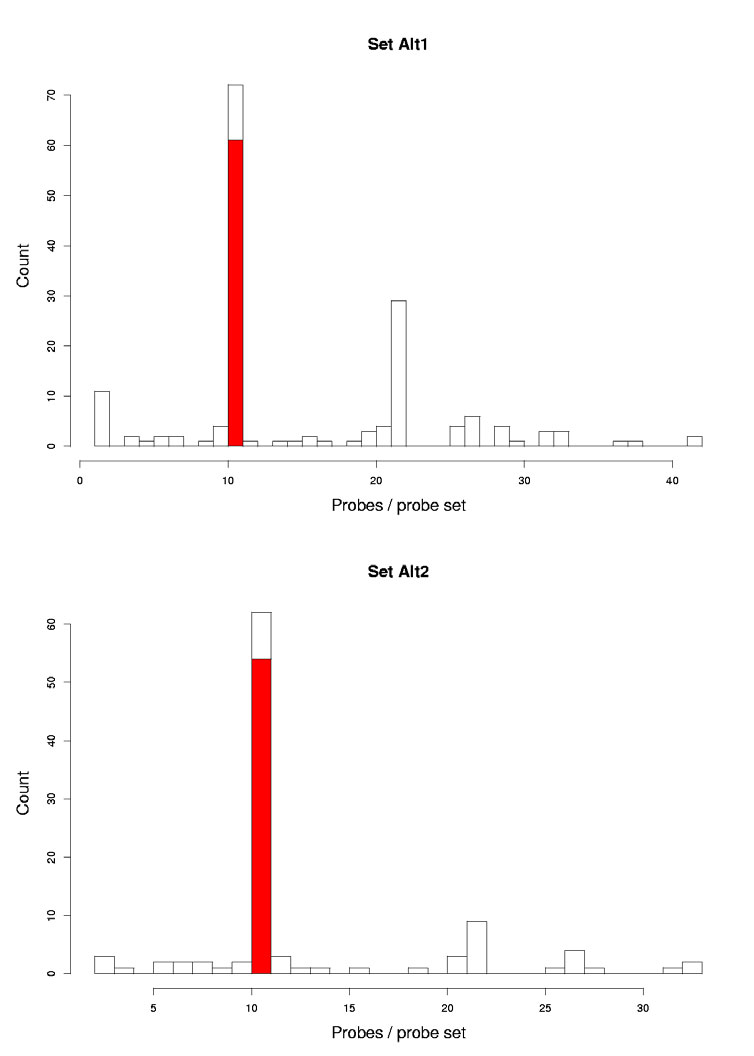
**Histograms of the number of probes per probe in the SDEGs. **Distribution of the number of probes per probe set in the sets of significantly differentially expressed genes for the set Alt1 (top) and the set Alt2 (bottom). The probe sets for which an identical probe set could be found in the Affy set are represented in red.

However, there is also a significant fraction of probe sets for which no simple equivalence could be made. As shown when discussing the matches, the situation is rather complex and a detailed examination for each case would be needed before a comparison between the mappings is possible. The presence of mismatch probes in the *alternative *mappings does not appear to have much influence. A legitimate concern can be that some of the probe sets in the *alternative mappings *only contain one or two probes, therefore the results obtained with theses probe sets may be dubious. In fact, only very few of these probe sets contain a small number of probes, as shown in Figure 5. Moreover, the minimal acceptable number of probes in a probe set has been reported to be lower than the number of probes commonly used [11]. Validation of the results found with our *alternative mappings *will have to be done *in silico *through the curation of the mappings by expert annotators and experimentally with techniques like RT-PCR.

### Software for inter-exchange mappings

We present a complex situation, with new probe sets built on matches between NCBI's RefSeq reference sequences and the sequences of the short oligonucleotide probes found on commercial arrays. This would be of little practical use for the research community without an easy access to the data or the tools used to obtain them. The framework developed in the package 'affy', an open-source and documented collection of data structures and functions for the analysis of *GeneChip *oligonucleotide arrays at the probe level, is currently used by a growing number of researchers. We take advantage of the features it offers by providing *alternative mapping *objects that can be 'plugged in', and used instead of the original ones, whenever wanted. The Bioconductor package 'altcdfenvs' contains helping functions, and documentation explaining how to achieve this.

## Discussion

We performed a matching of the Affymetrix probes against the latest reference sequences from NCBI's RefSeq data bank. A number of probes appear to match a large number of reference sequences, hence to match a large number of transcripts. When analyzing a real data set with state-of-the-art processing methods, we observe that the outcome of an analysis can be influenced by inaccuracies in the probes mapping. This is a potential problem when more and more people use 'high-throughput' procedures, to select 'significantly important genes' in an automated or semi-automated fashion.

We introduce an alternative mapping between probes and identifiers, our identifiers being NCBI's RefSeq IDs, and offer for download alternative mappings for the Affymetrix chips HG-U95Av2 and HG-U133A.

The *affy *package is a free environment to work with *Affymetrix *data at the probe level. It has capabilities to include alternative mapping and use them in a simple way.

Our work aims at showing potential problems. Biological expertise remains necessary to discuss the exact nature of each match. *Affymetrix *offers at their NetAffx web site [12] a tool that allows visualization of probes matching to multiple sequences. As the annotation of the human genome improves over time, our environment allows to update the probe-to-gene mapping accordingly and analyze microarray data using the best biological data and knowledge available.

The environment for alternative mappings we present could also be of use when using *GeneChips *designed for a certain organism with mRNA known to differ (a mutant or a slightly different organism for example).

## Conclusions

We built new 'probe to gene' mappings by matching the sequences for the probes found on human Affymetrix GeneChip arrays against NCBI's RefSeq database of sequences. Solely observing the distribution of the probes matching reference sequences, and comparing the results with the mapping provided by the manufacturer of the chips, we found potential problems such as probes matching several reference sequences and probes from different probe sets in the official mapping matching all the same reference sequence. Depending on the mapping used, the outcome of the data analysis will change as different genes may be selected as differentially expressed. We suggest that a good mapping of probe to genes changes in time, follows the most recent updates in sequence databases (the databases of sequences are constantly modified, with sequences corrected, and sometimes hypothetical gene sequences withdrawn). We like to picture the current situation, where the mappings are frozen, as a Dorian Gray-like syndrome: the apparent eternal youth of the mapping does not reflect that somewhere the 'picture of it' decays. We developed a set of open-source tools, perfectly integrated to an already existing working environment for Affymetrix arrays. The tools are documented and made available to the research community. They let one reproduce our results, and build other mappings.

## Methods

### Reference sequences and probe sequences

The database of reference sequences used for remapping the Affymetrix chips is NCBI's RefSeq (first release of NCBI's RefSeq, dated June 30th, 2003), freely available for download on the RefSeq website [13]. Only sequences tagged as *Homo sapiens *mRNA are considered.

The Affymetrix chip types HG-U95Av2 and HG-U133A, both designed for the study of the human transcriptome, were used. While similar features were observed for both, we focus on the HG-U133A to describe our results, as this is the chip with the most recent design.

The probe sequences for the chips are freely available on the Affymetrix website [14], as well as on the Bioconductor website [15] as meta-data packages. In total, there are 495930 probe sequences on the chips of type HG-U133A (245965 perfect match probes and 245965 mismatch probes).

### Probe matching

*Affymetrix GeneChips *have several probes per probe set, and a probe set usually represents a gene. Each probe is 25 bases long, and on most arrays probes are grouped in pairs. A probe pair is constituted of a perfect match (pm) probe, designed to match perfect a target gene sequence, and a mismatch (mm) probe, designed to measure non-specific hybridization. The mismatch probe differs from its associated perfect match probe only in the 13th base. In some cases, a probe set represents a fragment of a gene, e.g, 3-prime and 5-prime extremities of the same gene are used as internal control for the efficiency of the reverse transcription. We prefer the term 'probe set ID' to 'gene' since probe sets are not always genes. We call the association 'probes – probe set ID' a *mapping*. We refer to the grouping of probes in probe sets given by *Affymetrix *as the *official mapping*, while a grouping of the probes matching a reference sequence into a probe set is referred to as an *alternative mapping*. The short length of the probes, as well as several authors reporting a successful use of perfect matches only [3-5,16], suggest that the hybridization signals coming from exact matches alone are able to capture the expression signal reliably. We consider all the probes (including *mm*) as potential *pm *probes to perform the matching. The matching of a probe to a sequence is done using the Bioconductor package *matchprobes*. It performs an exact string matching, as done by the standard C library *string: *only complete sequence identity between a probe and a fragment of a reference sequence is counted as a match.

### Experimental data

We collected pancreatic tumor tissue from 8 patients and normal pancreatic tissue from 5. Tumor samples were collected from patients undergoing surgery for pancreatic tumors, quick frozen in liquid nitrogen and kept at -80°C until RNA extraction (the study was approved by the ethical committee for Copenhagen). The tissue was homogenized using a Polytron (kinematica. AG, Littau-Luzern, Schwitzerland). 5 μg RNA was extracted from each sample and labeled. The RNA was extracted according to the Trizol protocol (Invitrogen). The samples were applied to Affymetrix HG-U133A GeneChips according to manufacturer's instruction. Clinical results from this study will be published elsewhere (manuscript in preparation).

### Data processing

The probe level data are pre-processed using the affy software package [17]. No background correction is performed, and probe level intensities are normalized using the *vsn *[18] normalization method. Summary expression indexes are computed using the method *medianpolish*, using only perfect match probes. The exclusive use of perfect match probes is needed to have an identical pre-processing step for the *official mapping *and *alternative mappings*, to allow comparison of the results obtained.

### Availability

All the datasets and software used are freely available, and on a wide range of platforms (Linux, Microsoft Windows, MacOS X, other UNIX-like operating systems).

The original material used to obtain our results, software and data, is available from the web page: Alternative mappings .

The package altcdfenvs, new with the Bioconductor release 1.4 of May 2004 and available on the Bioconductor website, is designed to help researchers to build their own alternative mappings, using their own set of reference sequences and the probe sequences for the Affymetrix chip of their choice.

## Authors contributions

LG conceived, developed and implemented the concept of alternative mapping for Affymetrix chips, and drafted the manuscript. MM and LFH provided original experimental data. SK provided guidance with the choice of a source for reference sequences, and with comments and input on the manuscript.

## Figures and Tables

**Table 1:** Number of probe sets Number of complete probe sets (see Figure 3, top) in common between the sets of significantly differentially expressed genes (SDEGs) Affy, Alt1 and Alt2 (163, 103 and 103 probe sets respectively).

	Affy	Alt1	Alt2
Affy		61	54
Alt1	61		95
Alt2	54	95	
